# The Role of Terroir on the Ripening Traits of *V. vinifera* cv ‘Glera’ in the Prosecco Area

**DOI:** 10.3390/plants13060816

**Published:** 2024-03-12

**Authors:** Nicola Belfiore, Alessandra Amato, Massimo Gardiman, Federica Gaiotti, Sara Zenoni, Giovanni Battista Tornielli, Marianna Fasoli, Luigi Bavaresco

**Affiliations:** 1 CREA, Council for Agricultural Research and Economics, Research Centre for Viticulture and Enology, 31015 Conegliano, Italy; massimo.gardiman@crea.gov.it (M.G.); federica.gaiotti@crea.gov.it (F.G.); 2 Department of Biotechnology, University of Verona, 37134 Verona, Italy; alessandra.amato@univr.it (A.A.); sara.zenoni@univr.it (S.Z.); giovannibattista.tornielli@univr.it (G.B.T.); 3 Department of Sustainable Crop Production–Viticulture and Pomology Section, Università Cattolica del Sacro Cuore, 29122 Piacenza, Italy; luigi.bavaresco@unicatt.it

**Keywords:** terroir, grapevine, metabolomics, transcriptomics, wine, Prosecco

## Abstract

The grapevine (*Vitis vinifera* L.) is widely cultivated worldwide owing to the substantial commercial value of the grapes and other products derived from their processing, wines in particular. The grapevine is characterized by a remarkable phenotypic plasticity within the same variety, which shapes the final berry quality attributes hence reflecting the complex interactions between the plant and the environment leading to the expression of wine typicity. In this study, we explored the metabolomic and transcriptomic basis of the plasticity of Glera, a white berry grapevine variety particularly renowned for the production of wine Prosecco. The two selected vineyards varied for site altitude and pedoclimatic conditions. We highlighted that these environments determined different berry ripening dynamics at the level of both technological parameters and the total abundance and intrafamily distribution of phenolic compounds. Moreover, a clear impact on the grape aroma profile was observed. The genome-wide gene expression analysis of the berries revealed remarkable differences in the ripening transcriptomic program, reflecting the differences in water status, light exposure, and temperature experienced by the plants while growing at the two sites. Overall, this survey portrayed how the quality attributes of the cv ‘Glera’ grape berries may be affected by different environmental conditions within the typical area of Prosecco wine production.

## 1. Introduction

The white winegrape variety Glera (*Vitis vinifera* L.) is mostly grown in Northeastern Italy, under different pedo-climatic conditions. It was historically known under the name “Prosecco” until 2011 when the establishment of the Prosecco PDO (Protected Denomination by Origin) that disallowed utilizing it for the grape variety. The first records trace back to the 18th century, when historical documents [1] located the Prosecco variety in “Colli Berici” growing area (Vicenza province, Veneto Region), and to the 19th century on Conegliano hills as described in the Ampelography of Treviso province in 1870. The demand for this grape variety has grown over the years in particular for the production of sparkling wines based on the Martinotti method that was established by the pioneer Antonio Carpenè [2]. Nowadays, Prosecco bottles amount to about one billion/year worldwide. As a result of the flourishing Prosecco market, the area planted under Glera has increased considerably in recent years exceeding the 40,000 hectares cultivated in the Prosecco production area (Veneto and Friuli Venezia Giulia regions). In the Veneto region, it is now the most widely planted variety and accounts for over a third of the total area under vines.

Glera is known for being a particularly productive variety and for exhibiting quite high vigor. The phenology spreads along extended growing seasons, featuring a fairly early vegetative development and late harvest dates with the production of grapes at middle sugar and acidity level and considered semi-aromatic [3].

Terroir is a typical concept of the wine world that was defined by OIV (Resolution 333/2010) as follows: “Vitivinicultural terroir is a concept which refers to an area in which collective knowledge of the interactions between the identifiable physical and biological environment and applied vitivinicultural practices develops, providing distinctive characteristics for the products originating from this area. Terroir includes specific soil, topography, climate, characteristics and biodiversity features”. The effects of the terroir are mainly linked to the interaction between the environmental conditions and the cultivation practices that play a clear role in influencing the physiology of the vine therefore the quality of the grapes (i.e., the aromatic characteristics; [4,5]) in relation to the varietal behavior and response in term of plasticity. It is well known that grapevine varieties modify their performance in distinct environments, with some varieties (such as Cabernet Sauvignon and Chardonnay) offering more consistency and others (such as Sangiovese, Nebbiolo and Pinot Noir) showing greater variation [6].

The influence of the terroir factors on Glera grapevines cultivated in the Conegliano Valdobbiadene Denomination area was studied focusing on the effects on the plant physiology and the viticultural and enological parameters [7,8,9,10], and the role of soil was also dissected [11]. Molecular approaches have been also applied to characterize the relationship between the geographic origin and the genetic variability of Glera clones [12]. In recent years, omics approaches have been used to unravel the phenotypic plasticity of grapevine on a broad scale and to dissect the interactions between genotype and environment (i.e., vintage, location) during the course of berry development [6,13,14,15,16]. The berry ripening transcriptomic program of Glera was included in the study of five red and five white Italian grapevine varieties, selected from different winegrowing regions to represent diverse agronomic traits and different environmental adaptations [17]. Transcriptional changes during berry ripening were compared when the 10 varieties were grown under the same environmental conditions creating the opportunity to distinguish the core transcriptomic traits from those dependent on the berry skin color and genotype-specific features. In this study, the investigation context has switched and the transcriptomic changes of the Glera ripening berry were characterized to include the effect of the cultivation site. A similar characterization of the phenotypic plasticity of a white variety was previously performed for cv Garganega, where changes in the berry transcriptome were ascribed to different pedoclimatic conditions at four cultivation sites [15].

The goal of this survey is to assess the link between viticultural and enological aspects of Glera, enriched with the berry transcriptomic information, and two different terroirs in the Prosecco wine production area.

## 2. Results

### 2.1. Pedoclimatic Features of the Sites

**Figure 1:**
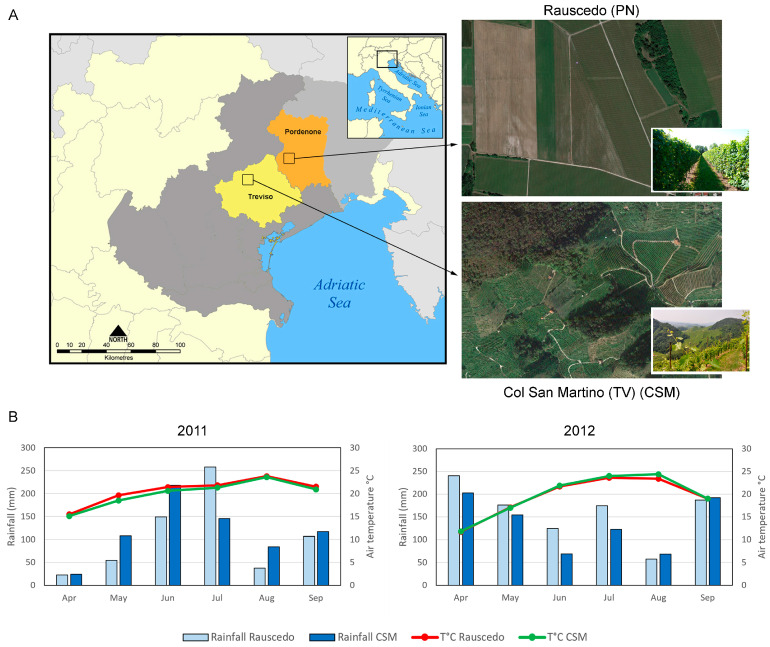
Sites, landscape, and environmental parameters. (**A**) Two vineyards were chosen both located in Northeastern Italy: one in the plain area of the PDO Prosecco in Rauscedo (Pordenone province; 46°02′78″ N; 12°80′87″ E); the other in the hilly area of the Conegliano Valdobbiadene-Prosecco DOCG district, located in Col San Martino (CSM; Treviso province; 45°89′78″ N; 12°06′22″ E). (**B**) Rainfall (mm) and average air temperature (°C) detected at the experimental sites during the 2011 (top panel) and 2012 (bottom panel) seasons.

*Vitis vinifera* cv ‘Glera’ berries were collected in the 2011 and 2012 growing seasons from two different vineyards, Rauscedo and Col San Martino (CSM), located in Northeastern Italy (Figure 1A). The physical and chemical characteristics of the soils are provided in Table 1. Sand and silt represented the most abundant granulometric fraction and are the components characterizing the texture of both soils. Both soils showed subalkaline pH values and high levels of active limestone, as opposed to low levels of total nitrogen and organic matter. The content of other compounds responsible for chemical fertility turned out to be high (K, Mg) in the CSM soil and low (K) or moderate (Mg) in the Rauscedo soil. The soil was shallower at CSM, with a maximum effective soil depth of 40 cm.

The meteorological conditions by rainfall and average temperature during the two growing seasons at the two sites are shown in Figure 1B. In 2011, the April-July rainfall was similar at both sites (485 and 496 mm in Rauscedo and CSM, respectively), whereas the August-September period was less rainy at Rauscedo, thus determining an overall drier season at Rauscedo (630 mm) with respect to CSM (698 mm). At mid ripening (MR) and harvest (R), Huglin and Growing Degree Days (GDD) indexes were slightly lower at CSM compared to Rauscedo due to a slightly cooler climate during the first part of the season (Table 2). In 2012, the climatic conditions varied: the April-July period was wetter at Rauscedo (+150 mm), while in August and September the frequency of rainfall events was very similar at both sites. Overall, the total rainfall was higher at Rauscedo (960 mm) than at CSM (810 mm). The average temperature recorded during the growing season was similar between sites with Huglin index and GDD comparable at MR (Table 2). Only in August, the average temperature was one degree higher at CSM.

### 2.2. Stem Water Potential

**Figure 2:**
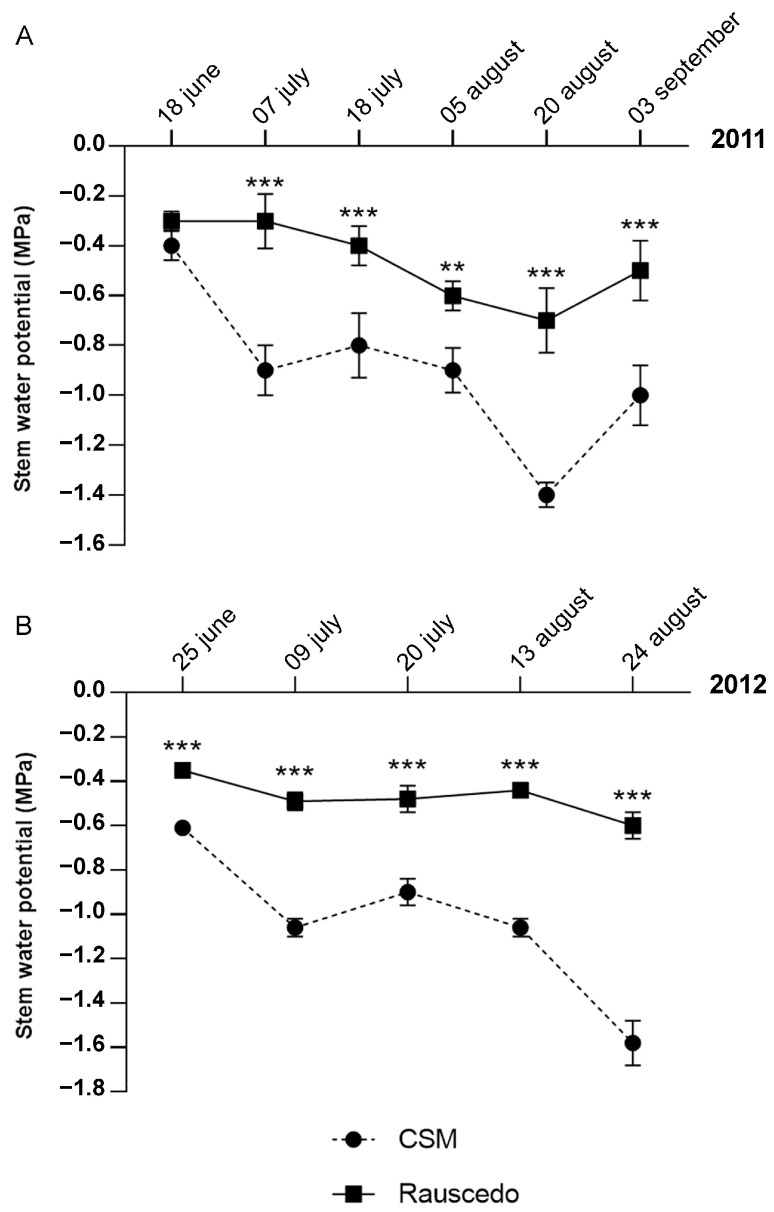
Water status assessment. Stem water potential was measured in 2011 (**A**) and 2012 (**B**) at the two locations. Asterisks indicate statistically significant differences (**, *p* value < 0.01; ***, *p* value ≤ 0.001; *t*-test). The error bars indicate the Standard Error of the mean (*n* = 10).

The evolution of the water potentials measured during the two tested years is shown in Figure 2. At Rauscedo, critical water levels were reached in neither season, as the seasonal water potential values averaged at −0.47 MPa, indicating lack of stress. The lowest value (−0.7 MPa) was recorded on 20 August 2011, confirming the gap from the critical threshold for water stress. At CSM, on the other hand, the seasonal averages were lower, reaching −0.9 and −1 MPa in 2011 (Figure 2A) and 2012 (Figure 2B) respectively, indicating the onset of moderate water stress. The water potential reached critically low values on 20 August 2011 (−1.4 MPa) and on 24 August 2012 (−1.6 MPa). Nevertheless, this extent of stress, especially when experienced in the final stages of the vegetative cycle, could result as beneficial in improving grape quality. Moreover, these conditions were usually transient showing an increase of the values by the next monitored date, like it happened for vintage 2011 when the water potential rose back to −1 MPa (3 September 2011).

### 2.3. Productive and Qualitative Parameters

Grape yield and quality parameters of both vintages were subjected to two-way ANOVA analysis (Table 3 and Table 4) that indicated a significant interaction between year and site for tartaric (*p* = 0.014) and malic acid content (*p* = 0.005). There was a significant main effect of the site for yield (*p* = 0.034), berry weight (*p* = < 0.001), tartaric (*p* = 0.007) and malic acid content (*p* = < 0.001), and of the year for number of bunches (*p* = 0.010) and berry weight (*p* = 0.001).

Vintage 2011 showed yield variation between the two sites, with CSM exhibiting lower production overall, vines producing fewer bunches per plant and smaller berries than Rauscedo. In 2012 the yield was similar between the two sites, but average berry weight was lower at CSM. In both seasons, the level of sugar, the total acidity and pH did not differ significantly between the two sites (Table 4). Tartaric acid levels recorded significantly greater values in Rauscedo for vintage 2012 (6.4 vs. 5.9), whereas malic acid concentration stayed greater in Rauscedo over both seasons (5.70 vs. 3.83 in 2011, and 6.07 vs. 3.03 in 2012) consistently with more suitable water conditions of the vines compared to the CSM location that limited the degradative metabolism of this organic acid.

### 2.4. Wine Sensory Analysis

The sensory characteristics of the wines were evaluated nine months after harvest.

The results of the two-way ANOVA on descriptive sensory analysis (Table S1) indicated a significant main effect for site on the aromatic lemon attribute (*p* = 0.017), the perception of pleasantness (*p* = 0.044), and the mouthfeel attribute of balance (*p* = 0.014). There were no significant effects (*p* < 0.05) of year and year x site interaction on any of the sensory attributes evaluated.

**Figure 3:**
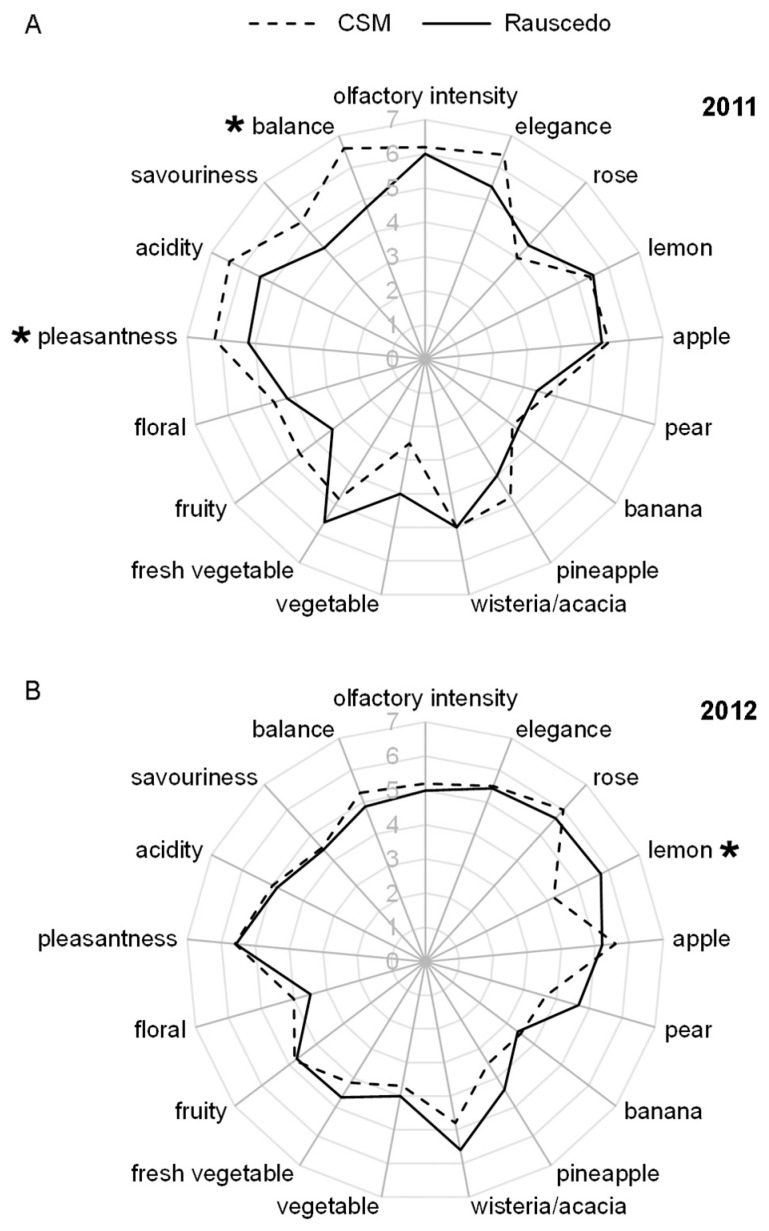
Descriptive sensory analysis of the wines produced in the two areas in the two vintages. The sensory profiles show differences by site (CSM, dashed line; Rauscedo, full line) and vintage (**A**), 2011; (**B**), 2012). Asterisks indicate statistically significant differences between sites (*p* value < 0.05).

The sensory profiles of the wines produced from the two sites in vintage 2011 were very similar in terms of aroma attributes, but the mouthfeel attributes of CSM were a slightly preferred over Rauscedo (Figure 3A). In the following season (2012; Figure 3B) location differences in aroma profile were more pronounced, particularly the lemon olfactory note scored slightly higher for Rauscedo (5.8 versus 4.2 for CSM). To investigate the molecular events at the basis of this significant difference in the aroma profile, we focused the analysis of berry metabolome and transcriptome variations by terroir on vintage 2012.

### 2.5. Metabolomic Profiling

**Figure 4:**
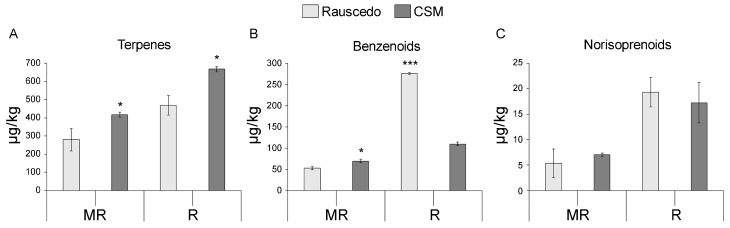
Aroma profiling of the ripening Glera berry. Total terpenes (**A**), benzenoids (**B**) and norisoprenoids (**C**) levels (µg/kg) were measured in the berries sampled at the two sites at MR and R developmental stages in the 2012 vintage. Asterisks indicate statistically significant differences (*, *p* value < 0.05; ***, *p* value ≤ 0.001; *t*-test). The error bars indicate the standard error of the mean (*n* = 3).

In order to detect correlations between the differences in wine aroma profiles by location and grape composition, 2012 berry samples were subjected to metabolomic profiling. The terpenic component, which is responsible of the citrus and floral notes, was the most abundant aromatic class detected at both sites and berry maturation stages. The greatest concentration was found at CSM, with significant differences between 140 and 200 units compared to Rauscedo, respectively at MR and R (Figure 4A). The greater content in terpene compounds at the CSM site is partly in line with what was found in a two-year study by Alessandrini et al. [9]. According to the authors, only in one year out of two the terpene fraction was more abundant at the highest altitude site. Benzenoids (responsible for vanilla, spicy and balsamic notes) were the second most abundant aromatic class and resulted significantly greater in CSM than Rauscedo at MR. This trend swapped at R when Rauscedo wines recorded 1.5-times greater values (Figure 4B). The least abundant class, with no significant differences between the two sites and phases, was represented by norisoprenoids (exotic fruit and tea notes; Figure 4C). The relative levels of aromatic compound classes are consistent with those reported in previous zoning studies [18].

**Figure 5:**
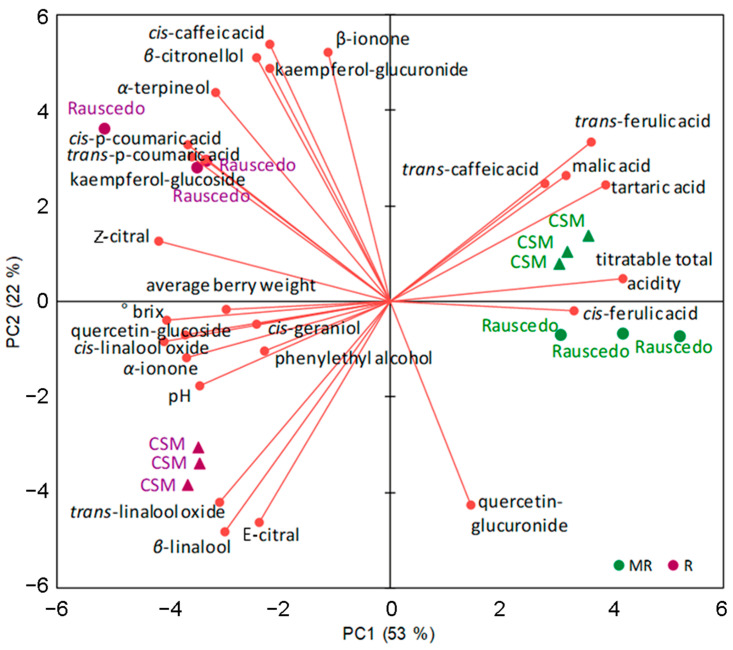
Growing site influence on the ripening Glera berry at compositional and metabolomic level. The PCA biplot—performed setting the content of berry metabolites and the main physical and chemical parameters (sugar, acidity, weight) as variables—shows clustering patterns of sites (CSM and Rauscedo) and ripening stages (MR, mid ripening shown in green dots/triangles; R, ripening shown in purple dots/triangles) in vintage 2012.

A detailed investigation of the volatile metabolites, hydroxycinnamoyl tartaric acids (HCTA) and flavonols was performed (Table 5 and Table 6). The data was explored by principal component analysis (PCA) that provided a clear differentiation between the grape maturation phases and the cultivation sites at harvest driven by the berry metabolite content and its physical and technological parameters (sugar, acidity, weight), which were set as variables in the multivariate analysis (Figure 5). The first component (PC1) accounted for the 53% of the variance and clearly separated the maturation stages, while the second component (PC2) accounted for the 22% of the variance and distinguished between growing sites that provided a minor contribution to the overall variance. The variables mainly associated with PC1 (squared-cosine higher than 0.5) were, as expected, linked to berry maturity (°Brix, pH, total acidity, tartaric and malic acid) and metabolites Z-citral, quercetina-glucoside, *cis*-linalool oxide, *α*-ionone, kaempferol-glucoside, *trans*-ferulic acid, *cis*-para coumaric acid, *trans*-para-coumaric acid, *cis*-ferulic acid, *α*-terpineol, *trans*-linalool oxide. The variables mainly associated with PC2 (squared-cosine higher than 0.5) were the metabolites *β*-ionone, *cis*-caffeic acid, *β*-citronellol, kaempferol-glucuronide, *β*-linalool. The PCA distribution evidenced that the two zones determined substantially similar berry compositions at MR with only minor variations. At R instead, samples clearly separated by geographical origin in relation to the aromatic compounds and flavonols content, while the effect of soluble solids was very small. Grapes harvested at CSM were characterized by highest levels of *trans*-linalool oxide, *β*-linalool and E-citral, while those harvested at Rauscedo had higher levels of *β*-citronellol, *β*-ionone, *cis*-caffeic acid, *cis*-p-coumaric acid, kaempferol-glucuronide and kaempferol-glucoside.

The volatile metabolite dataset was further investigated showing that at MR, the concentrations were greater at CSM, whereas at R only few compounds were found to be more abundant at Rauscedo (Table 5). A general increase in compound content was evident as ripening progressed. Exceptions were epoxylinalool and E-citral that, at Rauscedo, decreased 9.3 and 9.9%, respectively, while transitioning from MR to R. Overall, the synthesis of aromatic compounds was greater in Rauscedo than in CSM grapes, that exhibited a downward trend from MR to R stages for five metabolites, in particular *trans*-geraniol, menthol and *β*-citronellol, whose content respectively dropped 30.1, 29.1 and 26.7%. *β*-linalool and *trans*-geraniol (first and second most abundant among terpenes) represented together the 57% of the total terpene content at MR, while at R they accounted for the 53% at Rauscedo and the 70% at CSM. In particular, we assume that *β*-linalool was the major contributor to the differeces in the overall terpenic content evidenced in Figure 4. E-citral, epoxylinalool, *cis*-geraniol and geranic acid together did not exceed 2% of the total content, regardless phase, or cultivation site. The most abundant benzenoids at MR were benzaldehyde prevailing at Rauscedo with 36% of the class total, followed by phenylethyl alcohol (27%), while at CSM phenylethyl alcohol was the most abundant (45% of the total), followed by benzyl alcohol (26%). At R, methyl salicylate was the most abundant compound in Rauscedo grapes (76% of the total), while CSM showed concentrations of phenylethyl and alcohol benzaldehyde that each accounted for about 30%. Among the norisoprenoids, *α*-ionone was the most abundant, with values ranging from 69% in MR to 96% in R at CSM. Concerning HCTA and flavonols, at harvest the berries from CSM were less rich in *cis*-caffeic acid, *cis*-p-coumaric acid, *trans*-ferulic acid, 2-*S*-glutationil-caffeil-tartaric acid, kaempferol-glucuronide and glucoside (Table 6).

### 2.6. Transcriptional Profiling

**Figure 6:**
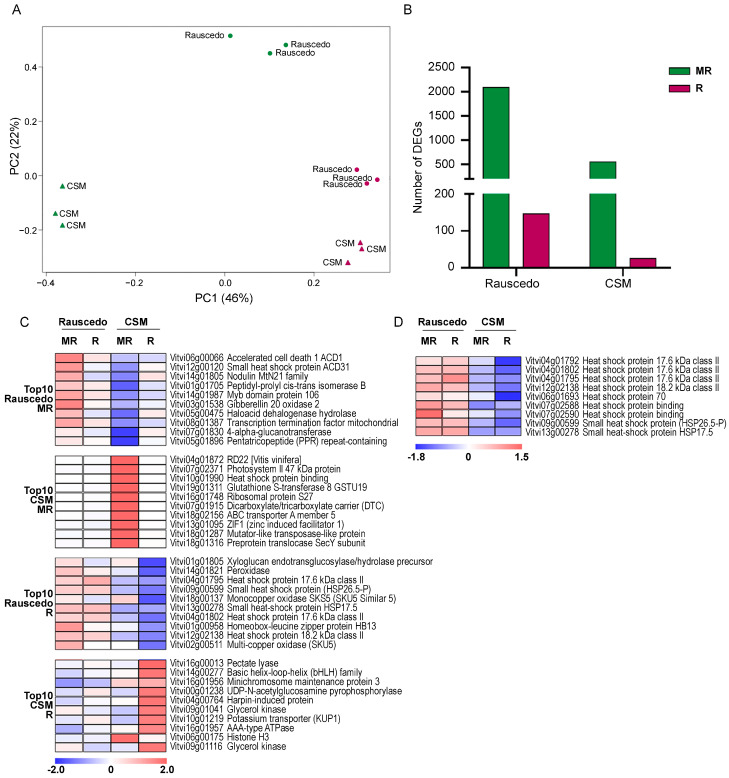
The plasticity of the ripening Glera berry transcriptome by vineyard site. (**A**) PCA score scatter plot of transcripts detected by microarray analysis in grape berries collected in 2012 during ripening in the two vineyards. (**B**) Number of differential expressed genes (DEGs) positively modulated in Rauscedo and CSM at the two ripening stages (MR, mid ripening; R, ripening). (**C**) Heat maps showing the expression patterns of the top-10 positively modulated genes over ripening at the two sites. The average expression values of three biological replicates are reported. (**D**) Heat map showing the expression pattern (over ripening and by site) of all the heat shock protein encoding genes resulted positively modulated at Rauscedo in mature samples. The average expression values of three biological replicates are reported.

To investigate the molecular events behind the variations in the metabolomic behavior, 2012 berry samples were also subjected to transcriptomic profiling. The investigation by PCA of the pericarp transcriptome dataset of the ripening Glera berries (Dataset S1) confirmed the consistency of the biological replicates (Figure 6A). PC1 explained 46% of the total variance and was attributed to differences in the ripening stage among the samples. PC2, explaining 22% of the total variance, mainly described differences between cultivation sites. Mature berry samples (at R) clustered together, whereas those collected at the MR distributed by growing location (Figure 6A). The plasticity of the ripening Glera berry transcriptome was investigated by *t*-test statistical analysis performed at both MR and R stages between Rauscedo and CSM vineyards (Dataset S1). A total of 2659 and 174 transcripts were significantly modulated (*p* < 0.01; |FC| > 2) at MR and R stage, respectively (Figure 6B), revealing that the Rauscedo site featured a greater gene modulation extent at both stages with respect to CSM. At MR, stage characterized by the highest gene modulation, 2100 genes were more expressed at Rauscedo and 559 at CSM (Figure 6B). The Gene Ontology (GO) enrichment analysis showed a significant enrichment of genes involved in the biosynthesis of secondary metabolites and in terpenoid-quinone biosynthesis among those up-regulated in Rauscedo (Supplementary Figure S1), whereas no significant enrichment resulted for genes up-regulated in CSM at this stage. This suggested that some ripening related genes were more activated in Rauscedo at MR in comparison to CSM, like unveiled by PCA. At R, we revealed that 147 genes were more expressed at Rauscedo and 27 at CSM (Dataset S1; Figure 6B). The GO enrichment analysis showed a significant enrichment of genes involved in pectin acetyltransferase activity and in water transporter/channel activity among genes up regulated in Rauscedo (Supplementary Figure S1). We reviewed the lists of the most modulated genes (according to the FC value) in the two sites at each stage and, as many were not annotated, focused on the top-10 most modulated genes with meaningful annotation and inspected their expression profile during ripening (Figure 6C). We revealed that genes upregulated in Rauscedo, in respect to CSM at MR, were similarly expressed at the ripening stage in the two sites. On the contrary, genes positively modulated in CSM were specifically highly expressed in this site in both developmental phases. Among the top-10 genes upregulated at the Rauscedo vineyard at ripening, we highlighted four genes encoding for heat shock proteins. We surveyed the whole (MR and R stages) expression profile at both sites of all heat shock protein resulted positively modulated at Rauscedo at R and then uncovered high expression since MR (Figure 6D).

To verify whether the berries responded to a specific environmental condition, we considered the temperature regimes of the two sites over the period right before harvest (i.e., one, three and five days before) and indeed observed that the average, minimum and maximum temperatures were higher at Rauscedo than CSM at each time frame considered (Table 7 and Table 8). A sudden drop in temperature affected the hillside area on 12 September, affecting the progression of CSM grape ripening, which reached the target maturity six days later. The daily temperature range in the five days preceding harvest (R) was greater at Rauscedo than CSM (between 3.5 and 4.9 °C difference), whereas the microclimate variations between the two sites at MR were minor. In Rauscedo, moreover, the average daily solar radiation during the growing season of April–September was about 1100 MJ/m^2^ higher than at CSM (19,800 vs. 18,700 MJ/m^2^). This means that during the 183 days considered, there was an over 200,000 MJ/m^2^ higher energy availability at the Rauscedo site, i.e., the energy of 10 days.

## 3. Discussion

The ripening phase is a crucial period that influences the composition of the grapes and therefore of the wine, and during which physical (weight, volume, color, and softness) and chemical (pH, acidity, sugars, phenolic and aromatic composition) changes take place. The environmental and climatic conditions, as well as the agronomic approach, influence the changes that occur within the same grape variety.

The two sites studied in this work differed mainly in altitude, exposure (south and west-facing at CSM and Rauscedo, respectively), water availability (absent at CSM), soil texture, effective soil depth (greater at Rauscedo) and solar radiation (greater at Rauscedo). The variation of these key factors determined the expression of the two terroirs.

Regarding the technological aspects, the grape soluble solids content of both vineyards was in line with the requirements by the respective production regulations to achieve a minimum alcohol level between 9.0 and 9.5% by volume depending on the wine style. Total acidity also exceeded the minimum requirement of 4.5 g/L set by the production regulations.

The estimated crop load varied between the monitored vineyards likely due to the influence of irrigation on the Rauscedo vines that presented greater vigor and higher Ravaz values, especially in 2012. The Ravaz index at CSM in 2012 was slightly lower than the typical value for the area (~6.5) although showing expression of vegetative-productive balance. In both vintages, also the yield was significantly impacted by water availability as lower water potential values were measured at the hilly site (CSM) compared to the plain site of Rauscedo, also remarking the influence of site orography, soil stratigraphy and depth. In 2011, the number of bunches per plant was significantly lower in CSM likely contributing to the drop in production compared to Rauscedo. Overall, the production and quality values determined in this trial at CSM were in line with those highlighted in a previous zoning study in the same area [18].

Water availability dissimilarities between cultivation sites seemed to have influenced the monoterpenes content, in step with previous studies reporting increased concentrations of monoterpenes such as linalool and geraniol under mild to moderate water stress conditions [19,20,21,22] likewise at the CSM vineyard. This response was seen associated with increased expression of terpenoid synthase genes [19,23].

Although the average temperatures at the two locations were similar throughout the season, Rauscedo recorded consistently higher temperatures than CSM in the five days prior to harvest: the mean and maximum temperature values were respectively augmented 3.5 and 7 °C. This situation could have led to a reduced synthesis of terpene compounds at Rauscedo. In fact, we hypothesize that high temperatures during ripening can reduce the synthesis and accumulation of aromatic compounds, especially the more thermolabile ones: cooler vintages and areas promote a more gradual increase of total terpenes during ripening compared to warm areas, which favors the achievement of greater content at maturity [24,25].

At Rauscedo, ambient light availability was always higher than at CSM (+200,000 MJ/m^2^) throughout the growing season, including the last days before harvest. This difference may have negatively impacted the final concentration of total terpenes and linalool at Rauscedo, in line with the reports of terpenoids sensitivity to sunlight in Muscat varieties [26,27,28], Sauvignon Blanc and Riesling [29]. Moreover, Rauscedo vines exhibited narrower leaf walls expositing the bunches to extra sunlight, especially during the period between trimming and canopy recovery (first ten days of June and August), and this condition may also have contributed to a reduced terpenol synthesis and/or an increase in linalool degradation with respect to CSM. On the contrary, the synthesis of kaempferol is regulated by a light-induced transcription factor [30], and greater levels of this compound were indeed reached in the Rauscedo grapes, which benefited of higher light intensities in the pre-harvest period compared to CSM.

Vintage 2012 showed more pronounced differences in the aroma profiles, in particular the lemon olfactory note that was mentioned for Rauscedo grapes. This could be partially explained by the citrusy lemon note of β-citronellol, whose concentration in the berries was more than double in Rauscedo compared to CSM (14 µg/kg versus 6 µg/kg), although the total terpene content was lower in the grapes grown at Rauscedo than at the CSM hillside site.

To understand the molecular basis of the environmental impact on ripening Glera berries, we explored the transcriptomic changes over vintage 2012 of two vineyards selected to maximize environmental differences (site altitude in particular) and minimize variations in the agricultural practices, such as the training system, row orientation, planting layout, vineyard age, and rootstock. The investigation of transcriptomic data by PCA revealed that the berry ripening program dynamics differed by growing site. The sample distribution explaining the greatest variance evidenced that PC1 described shared molecular changes associated with berry development, as reported in previous transcriptome surveys [13,31,32,33,34], whereas PC2 highlighted unique behaviors accounting for the highly plastic responses of Glera berries to the pedoclimatic conditions at the two growing sites. We observed that samples collected from the two vineyards at the MR phase were clearly separated, while mature samples, albeit still distinguishable, were more like each other. Indeed, we found more differentially expressed genes between vineyards at MR than at R, that however confirmed that location influence on the overall grape berry development transcriptomic program as previously reported [13,15,35].

In our investigation we explore genes specifically modulated for each vineyard at both ripening stages. CSM vineyard featured the least number of modulated genes during berry maturation but site-specific, especially at MR. Among these, we found the *Responsive to Dehydration 22* (*RD22*, Vitvi04g01872; [36]) and a heat shock protein (Vitvi10g01990) encoding genes that might be induced in response to the slightly higher temperature registered at CSM compared to Rauscedo at the MR stage.

The 2012 temperature conditions switched around the R stage, when the almost ripe grapes were subjected to significantly higher temperatures at Rauscedo compared to CSM and we speculate that this site-specific environmental variation is connected with the recorded up regulation of many heat shock protein encoding genes.

Besides triggering transcriptional changes related to cellular homeostatic responses, temperature was shown to impact the modulation of genes involved in the biosynthesis of volatile aromas [37] and, when significantly high, to distort the aromatic pattern by depleting terpenes in favor of benzenoids in post-harvest withering Corvina berries [38]. Consistently, we here recorded a significantly higher content of benzenoids in Rauscedo vineyard in comparison to that found in CSM at the ripening stage.

In line with metabolomic data showing a higher amount of terpenes in CSM than Rauscedo grapes at the R stage, we detected the up regulation of the gene *1-hydroxy-2-methyl-2-(E)-butenyl 4-diphosphate synthase* (*HDS*; Vitvi06g00286), involved in the monoterpenes biosynthetic pathways and recently shown to be upregulated in volatile-reach grapevine varieties [39]. In line with this, Rauscedo grapes at MR showed positive modulation of genes that were actually found the least expressed in varieties featuring aromatic traits [39] suggesting the reduced aroma-related transcriptomic layout of Rauscedo grapes, like the *Violaxanthin de-epoxidase* (*VDE*; Vitvi04g01082) and *Zeaxanthin epoxidase* (*ZEP*; Vitvi07g01745) encoding genes. *VDE* and *ZEP* are both involved in the violaxanthin cycle and were shown to increase in expression throughout exocarp development in the white cv Alvarinho [40], proposing the role of violaxanthin cycle in protecting the photosynthetic apparatus from damage during berry development and ripening. Moreover, the expression of *VDE* was shown to increase in light-exposed Sauvignon Blanc berries, compared to shaded ones [35]. Likewise, Rauscedo grapes responded to the higher radiation detected in this site compared to CSM during the 2012 season by involving the violaxanthin cycle—that we recorded as an increased expression of *VDE* and *ZEP* in Rauscedo grapes—to likely preserve the bunches from excessive light exposure.

Overall, this survey explored the metabolomic and transcriptomic basis of the plasticity of the cv ‘Glera’. We highlighted different berry ripening dynamics by vineyard site for technological parameters, abundance and composition of phenolic compounds, and grape aroma profile. The genome-wide gene expression analysis of the berries revealed remarkable differences in the ripening transcriptomic program, revealing the differential response of the vines to the pedoclimatic conditions within the typical area of Prosecco wine production.

## 4. Materials and Methods

### 4.1. Sites and Climate Description

Grapevine plants of *Vitis vinifera* cv Glera were grown in Northeastern Italy at two different vineyard sites: the plain territory of the PDO Prosecco in Rauscedo (Pordenone province; 46°02′78″ N; 12°80′87″ E), and the hilly area of the Conegliano Valdobbiadene-Prosecco DOCG district, located in Col San Martino (CSM; Treviso province; 45°89′78″ N; 12°06′22″ E).

The vines in the selected vineyards were 15 years old, grafted onto the Kober 5BB rootstock (Berlandieri × Riparia), and Sylvoz trained with three canes per vine with 10–12 buds each. The permanent cordon was positioned 1.3 m from the ground, and the planting distances were 3.0 m between rows and 1.2 m along the row, with a density of 2777 vines per hectare.

Vineyard management involved native cover crops with mowing during the spring-summer period and two topping operations between the end of flowering and the beginning of veraison. The fertilizer supply provided for the application in post-sprouting and post-harvest of 80 kg/ha of N and K, and 40 kg/ha of P per year. The vineyard in Rauscedo was irrigated with drip irrigation, while in CSM it was rainfed. The trial considers data recorded in 2011 and 2012.

### 4.2. Soil Analysis and Meteorological Data

Soil samples were taken at the end of the winter (before spring fertilization in 2011) at two different depths (0–30 and 30–60 cm). Five 1-kg subsamples were taken from each vineyard, mixed, and analyzed according to the official methods for soil chemical analysis [41].

The meteorological data were provided by the Veneto region meteorological services (ARPAV) for the CSM site and by the Friuli Venezia Giulia regional meteorological observatory (ARPA FVG, Pordenone, Italy) for the Rauscedo site.

Huglin’s heliothermal index was calculated using the formula:

$$\overset{30.09}{\underset{01.04}{\sum }}\left(\left(\mathrm{Tmean}-10\right)+\left(\mathrm{Tmax}-10\right)\right)/2\times K$$

where: Tmean = Average daily temperature

Tmax = Maximum daily temperature

K = coefficient of latitude (1.04)

GDD = average daily temperature −10 °C

### 4.3. Productive Parameters

For both vintages, yield, number of bunches per vine, and average bunch weight were determined at harvest on 15 representative plants. The harvest dates were as follows: 1 September 2011, at Rauscedo—89 days after flowering (DAF); 6 September 2011, at CSM—88 DAF; 11 September 2012, at Rauscedo—102 DAF; 18 September 2012, at CSM—104 DAF. Pruning weights were recorded during the winter, allowing to calculate the Ravaz index (yield/pruning weight).

### 4.4. Stem Water Potential

Stem water potential was measured with the Scholander pressure chamber (model 600; PMS Instrument Company, Albany, OR, USA) between 18 June and 3 September in 2011 and between 25 June and 24 August in 2012. Measurements were taken between 13:00 and 14:30.

At each site and for each day of measurements, one healthy and undamaged leaf was chosen between the 4th and 6th node after the last cluster of a middle shoot of the fruiting branch of each of ten selected vines [42].

Prior to the analysis, the leaf was placed in a black aluminum-coated bag for one hour [43,44] to promote stomatal closure, stop transpiration and theoretically equilibrate the lymph flow of the petiole with the plant and the water content of the soil. The value of the pressure at which the first drop of lymph flowed out was taken as the value of the water potential.

### 4.5. Grape Sample Collection

Average berry weights, soluble solids, pH, titratable acidity, malic, and tartaric acids were determined from a pool of 100 berries randomly picked from the harvested grape mass in 2011 and 2012. Three biological replicates were created per sample.

Grape berries for metabolomic and transcriptomic analysis were taken in 2012 at two different ripening stages: at Mid ripening stage (MR) when berries were translucent (BBCH 83), and at harvest (Ripening stage, R; BBCH 89). Aromatic analysis and the determination of flavonols and hydroxycinnamoyl tartaric acids (HCTA) were based on a collection of 80 and 20 berries respectively, whereas 50 were processed for transcriptomic analysis. Three biological replicates were created from both cultivation site and at each stage, collecting berries with pedicels from the top, central, and bottom portions of clusters selected at both row sides (i.e., sun-exposed and shaded). Samples were flash-frozen in liquid nitrogen and then stored at ultra-freezing temperatures (−84 °C) until processing.

### 4.6. Chemical Analysis

Soluble solids were determined on the must by refractometry at 20 °C with a digital refractometer (ATAGO PR-32) and expressed in Brix degrees, while titratable acidity (g/L) and pH were measured on 20 mL of must at 20 °C with a microtitrator (Crison micro TT 2022—Crison strumenti SPA, Carpi, Modena, Italy), equipped with a pH electrode (Hamilton FlushTrode P/N 238060/08), using a 1 N NaOH titrant solution (SodiumHydroxide ACS reagent Honeywell Fluka 30620).

Malic and tartaric acid content (g/L) was determined using the RP-HPLC method (Agilent 1220 Infinity LC; Agilent Technologies, Santa Clara, CA, USA) on must samples diluted 50 times and filtered following the protocol proposed by Kordi et al. [45].

The glycosylated aromas (terpenes, norisoprenoids, and benzenoids) were determined according to the method reported by Vrhovsek et al. [46]. In brief, solid phase extraction (SPE) was performed using ENV+ cartridges 1 g (Biotage, Uppsala, Sweden), the free aromatic compounds were eluted with 30 mL of dichloromethane and the bound aromatic compounds with 30 mL of methanol; the latter was then treated with a AR2000 pectolytic enzyme. GC analysis was performed using a Trace GC Ultra gas chromatograph coupled with a TSQ Quantum Tandem mass spectrometer (Creative Proteomics SUITE 115, Shirley, NY, USA). GC separation was performed on a 30 m VF-WAXms capillary column with an internal diameter of 0.25 mm and a film thickness of 0.25 m (Varian, Inc., Palo Alto, CA, USA).

Flavonols and hydroxycinnamoyl tartaric acids (HCTA) were determined by HPLC analysis on samples of 20 still-frozen berries, according to Di Stefano and Cravero [47]. Analyses were performed on the supernatant obtained from the pulp and skin samples. Chromatographic separation of HCTA and flavonols from the skins was performed using a ThermoHypersil-Keystone ODS Hypersil RP C-18 column (Thermo Scientific, Waltham, MA, USA) [48].

### 4.7. Micro-Vinification and Wine Sensory Analysis

At each site, 150 kg of grapes were collected from three groups of ten vines in different parts of the vineyard and taken to the cellar for micro-vinification. The berries were crushed and pressed using a membrane press operating at 1.2 bar. The must was then mixed with three mg L^−1^ of pectolytic enzyme and 100 mg L^−1^ of potassium metabisulphite. After a clarification period of 12 h, the juice and the lees were separated. Alcoholic fermentation took place at 18 °C for 18 to 20 days. At the end of February of the year following the harvest, the wine was filtered and clarified again before being bottled.

The sensory analysis of the wines was carried out each year in June by the same seven trained tasters. The tasting panel was formed by 7 people, all belonging to a sensory group with long experience in wine tasting, composed of oenologists, winemakers, and sommeliers. Seventeen attributes were considered: eleven aroma-related attributes (olfactory intensity, elegance, rose, lemon, apple, pear, banana, pineapple, wisteria/acacia flower, vegetable, fresh vegetable), three mouthfeel attributes (acidity, savouriness, balance) and three final perception characteristics (fruity, floral, pleasantness). The different attributes were quantified using a ten-point intensity scale [49]. For each sample, the judges received a 30 mL sample served at 18 ± 1 °C in glasses covered to prevent loss of volatiles. The order of presentation was randomized between judges and sessions.

### 4.8. RNA Extraction and Microarray Analysis

Total RNA was isolated from 200 mg of ground berry pericarp using the Spectrum™ (Spectrum Chemical, New Brunswick, NJ, USA) Plant Total RNA kit with modification according to Fasoli et al. [50]. The RNA quality was determined using a NanoDrop 2000 spectrophotometer (Thermo Fisher Scientific, Waltham, MA, USA) and the quantity was assessed using a Bioanalyzer Chip RNA 7500 series II (Agilent, Hong Kong, China). The cDNA synthesis, labelling, hybridization, and washing steps were conducted according to the NimbleGen Arrays User’s Guide (v3.2). Each hybridization was performed on a NimbleGen microarray 090818 Vitisexp HX12 chip representing 29,549 predicted grapevine genes covering approximately 98.6% of the genes predicted in the V1 annotation of the 12X grapevine genome. Microarray chips were scanned, and images were analyzed as described by Dal Santo et al. [13]. Reported values in figures and dataset represent the means of three replicates and a Pearson’s correlation analysis was carried out to evaluate the robustness of the replicates. A gene was considered to be expressed if the normalized expression value was higher than the average value of chip negative control present in at least two of the three replicates. Only genes with an average fluorescence value >150 in all the conditions were considered.

### 4.9. Statistical Analysis

A two-way fixed-effects ANOVA with interaction was performed to evaluate the effects of site and vintage (year) on yield, grape quality parameters, and wine sensorial evaluation, followed by one-way ANOVA (Tukey’s test, *p* ≤ 0.05) for multiple comparisons of means when a statistically significant interaction was not found, using STATISTICA v.7 software (statSoft Inc., Tulsa, OK, USA). A one-way ANOVA analysis was applied for each individual year. Residual analysis was performed to test for the assumptions of the ANOVA. Normality was assessed using Shapiro-Wilk’s normality test and homogeneity of variances was assessed by Levene’s test. There were no extreme outliers, residuals were normally distributed (*p* > 0.05) and there was homogeneity of variances (*p* > 0.05).

Due to the different timing of observation and the different number of sampling points in the two years, the stem water potential data were analyzed with a one-way ANOVA for each individual vintage.

A principal component analysis (PCA) was applied on the standardized and normalized data of the productive and qualitative parameters, and on the transcriptomic dataset, using XLSTAT 2022.1.1 software (statistical and data analysis solution, Paris, France) aiming at exploring the variance of the datasets in unsupervised manner and unveiling hidden connections and trends between variables. Bartlett’s test and Pearson’s correlation were used in advance to check, respectively, the homogeneity of the variances and the significance of the correlations between the variables.

The Kaiser-Meyer-Olkin test (KMO test) was applied to check the adequacy of sampling and variables with value less than 0.5 were rejected. Only variables with a factor/variable correlation value greater than 0.65 were included in the analysis, and the cosine-squared value (>0.5) was considered to interpret the link between the variables and the dimensions (PC1 and PC2).

Differentially expressed genes (DEGs) were determined by performing a *t*-test using TMeV software (V4.9.0; https://webmev.tm4.org/about accessed on 11 January 2024) with a *p*-value (*p*) of 0.01% and then filtered by applying a fold-change (FC) threshold of >2 or <−2 (Dataset S1). Genes that were significantly modulated during ripening in the two vineyards and between vineyards at each stage, were extracted.

The Gene Ontology (GO) enrichment analysis was performed by using the ShinyGO v.0.741 software [51] with a False Discovery Rate (FDR) cutoff of 0.1.

## Figures and Tables

**Table 1:** Main physical and chemical characteristics of the soil at the two experimental sites. Extraction details and methodologies are reported.

Soil Feature	Rauscedo	CSM
Texture	Silt loam	Loam
Sand (%)	31	45
Silt (%)	59	32
Clay (%)	10	23
Organic matter (%) (Walkley-Black)	1.20	1.40
pH (in water 1:2.5)	8.33	8.43
Active lime (CaCO_3_) (%)	14.5	15.0
Available P (ppm) (Olsen)	11.6	12.8
Total N (%) (Kjeldal)	0.02	0.05
Exchangeable K (ppm) extracted with BaCl_2_	100	380
Exchangeable Ca (ppm) extracted with BaCl_2_	1214	2740
Exchangeable Mg (ppm) extracted with BaCl_2_	114	307

**Table 2:** Cumulative growing degree days (GDD, base temperature 10 °C) and Huglin index recorded at the two sites in 2011 and 2012. MR, mid-ripening; R, ripening.

	2011			2012		
	GDD_10_ at MR (°C)	GDD_10_ at R (°C)	Huglin Index (°C)	GDD_10_ at MR (°C)	GDD_10_ at R (°C)	Huglin Index (°C)
Rauscedo	1400	1611	2660	1380	1599	2410
CSM	1327	1581	2510	1403	1707	2412

**Table 3:** Two-way ANOVA results on yield and grape quality parameters at ripening (2011 and 2012) at the two sites. Significant values are indicated in bold (*p* value < 0.05; two-way ANOVA).

Variable	*p*-Value		
	Year	Site	Year × Site
Yield/vine (kg)	0.152	**0.034**	0.554
Bunches/vine (n.)	**0.010**	0.102	0.187
Bunch weight (g)	0.714	0.378	0.203
Berry weight (g)	**0.001**	**<0.001**	0.062
Ravaz index	0.893	0.108	0.176
TSS °Brix	0.733	0.470	0.470
Titratable acidity (g/L)	0.638	0.967	0.139
Tartaric acid (g/L)	1.000	**0.007**	**0.014**
Malic acid (g/L)	0.187	**<0.001**	**0.005**
pH	0.067	0.364	0.050

**Table 4:** Grape yield and quality parameters of in 2011 and 2012 vintages. Asterisks indicate statistically significant differences (*, *p* value < 0.05; two-way ANOVA).

2011	Yield/Vine (kg)	Bunches/Vine (n.)	Average Bunch Weight (g)	Average Berry Weight (g)	Ravaz Index	TSS °Brix	Titratable Acidity (g/L)	pH
CSM	7.1	25	283	2.3	6.9	16.1	6.9	3.21
Rauscedo	9.9	34	273	2.8	7.2	16.0	6.3	3.26
*p* value	0.036 *	0.020 *	0.815	0.012 *	0.864	1	0.202	0.164
**2012**	**Yield/Vine (kg)**	**Bunches** **/Vine** **(n.)**	**Average Bunch Weight (g)**	**Average Berry Weight (g)**	**Ravaz Index**	**TSS °Brix**	**Titratable Acidity (g/L)**	**pH**
CSM	9.0	38	241	2.1	5.8	16.2	6.1	3.28
Rauscedo	10.7	40	268	2.4	8.1	15.7	6.8	3.26
*p* value	0.226	0.736	0.057	0.026 *	0.055	0.284	0.108	0.069

**Table 5:** Terpenes, benzenoids and norisoprenoids levels (µg/kg) in the berries sampled at the two sites at mid-ripening (MR) and ripening (R) in vintage 2012. * *p* value ≤0.05; ** *p* value ≤ 0.01; *** *p* value ≤ 0.001 (*t*-test). Δ represents the percent difference between CSM and Rauscedo. Values in bold represent significantly higher content in CSM compared to Rauscedo.

Class	Compound	Scent Notes	MR		*p* Value	Δ (%)	R		*p* Value	Δ (%)
			Rauscedo	CSM			Rauscedo	CSM		
Terpenes	*trans*-linalool oxide	camphor	9.1	10.0	0.025 *	**10.0**	11.8	18.5	0.024 *	**56.6**
	*cis*-linalool oxide	coriander	12.1	18.4	0.010 **	**52.6**	20.9	23.3	0.097	11.6
	*trans*-geraniol	rose	54.3	148.7	0.002 **	**174.1**	96.9	104.0	0.974	7.3
	*β*-linalool	flower/rose	106.2	92.3	0.684	−13.1	150.7	363.0	0.000 ***	**140.8**
	menthol	peppermint	3.0	7.5	0.002 **	**148.8**	6.8	5.3	0.169	−21.6
	Z-citral	citrus	6.9	7.1	0.833	3.3	13.3	13.0	0.133	−2.2
	*α*-terpineol	jasmine/violet	28.2	37.7	0.127	33.7	53.3	37.7	0.009 **	−29.3
	E-citral	citrus/lemon	1.0	1.0	0.249	−6.6	0.9	2.0	0.016 *	**114.1**
	epoxylinalol	floral	1.1	2.1	0.309	79.9	1.0	2.2	0.128	110.5
	*β*-citronellol	Lemon/lemongrass	4.8	8.4	0.049 *	**74.0**	14.3	6.1	0.028 *	−57.1
	*cis*-geraniol	rose/citrus	0.4	3.1	0.001 ***	**646.0**	2.8	3.9	0.402	41.0
	rose-oxide	rose	8.3	10.9	0.227	31.6	14.3	9.7	0.124	−32.4
	geranic acid	dried flower	2.0	3.2	0.080	62.3	3.5	4.4	0.283	26.7
	*p*-menth-1-en-4-ol	pepper, spicy, citrus	14.7	16.6	0.132	13.4	51.9	23.5	0.185	−54.7
	isogeraniol	geranium	26.8	50.2	0.014 *	**87.2**	26.5	51.1	0.001 ***	**92.6**
Benzenoids	methyl salicylate	camphor	9.5	7.4	0.102	−22.3	210.2	22.8	0.000 ***	−89.2
	benzyl alcohol	cherry, dried fruits	9.1	18.0	0.000 ***	**98.1**	15.4	20.7	0.022 *	**33.9**
	benzaldehyde	bitter almond	18.9	12.5	0.125	−34.1	24.3	32.6	0.362	34.0
	phenylethyl alcohol	rose	15.0	31.4	0.000 ***	**108.6**	26.3	33.8	0.055	28.7
Norisoprenoids	*α*-ionone	tropical fruits, tea	5.1	5.0	0.933	−2.1	14.9	16.6	0.720	11.3
	*β*-ionone	violet/dried fruits	0.3	2.2	0.095	747.9	4.3	0.6	0.013 *	−85.2

**Table 6:** HCTA and flavonols levels (mg/kg) in the berries sampled at the two sites at mid-ripening (MR) and ripening (R) in vintage 2012. * *p* value ≤ 0.05; ** *p* value ≤ 0.01; *** *p* value ≤ 0.001 (*t*-test). Δ represents the percent difference between CSM and Rauscedo.

Compound	MR		*p* Value	Δ (%)	R		*p* Value	Δ (%)
	Rauscedo	CSM			Rauscedo	CSM		
*cis*-caffeic acid	0.22	0.29	0.097	32.0	0.31	0.23	0.001 ***	−24.8
*trans*-caffeic acid	4.25	6.32	0.003 *	48.7	4.04	3.50	0.312	−13.5
*cis*-p-coumaric acid	0.53	0.82	0.001 ***	54.6	1.38	0.98	0.012 *	−28.7
*trans*-p-coumaric acid	1.49	2.11	0.001 ***	42.3	3.11	2.35	0.056	−24.2
2-S-glutationil-caffeil-tartaric acid	0.23	0.16	0.003 **	−31.1	0.21	0.09	0.001 ***	−53.9
*cis*-ferulic acid	0.09	0.11	0.063	30.0	0.06	0.07	0.152	18.4
*trans*-ferulic acid	0.18	0.22	0.115	21.1	0.13	0.08	0.001 ***	−43.3
Quercetin-glucuronide	5.09	4.67	0.531	−8.3	3.63	5.00	0.121	37.8
Quercetin-glucoside	3.52	2.51	0.133	−28.6	9.71	9.91	0.915	2.1
Kaempferol-glucuronide	0.20	0.09	0.099	−54.3	0.58	0.09	0.002 **	−84.3
Kaempferol-glucoside	0.08	0.09	0.878	3.3	2.99	1.10	0.029 *	−63.1

**Table 7:** Microclimate at the two sites in the period immediately before the mid-ripening stage (vintage 2012), i.e., one, three, five days before mid-ripening.

Days before Mid-Ripening		1	1-3	1-5
Avg T (°C)	Rauscedo	25.1	25.6	25.4
	CSM	26.9	27.3	27.2
Min T (°C)	Rauscedo	18.6	18.4	18.2
	CSM	20.1	20.3	20.0
Max T (°C)	Rauscedo	32.6	33.7	33.5
	CSM	34.2	35.1	35.1
Daily temperature range (°C)	Rauscedo	14.0	15.3	15.3
	CSM	14.1	14.8	15.0
Solar radiation (MJ/m^2^)	Rauscedo	21,920	23,260	23,680
	CSM	21,727	21,204	21,915

**Table 8:** Microclimate at the two sites in the period immediately before ripening stage (vintage 2012), i.e., one, three, five days.

Days before Harvest		1	1-3	1-5
Avg T (°C)	Rauscedo	22.5	21.9	21.6
	CSM	18.8	18.4	17.7
Min T (°C)	Rauscedo	16.0	15.0	15.3
	CSM	13.5	12.6	12.3
Max T (°C)	Rauscedo	31.8	31.4	30.4
	CSM	24.5	24.1	23.8
Daily temperature range (°C)	Rauscedo	15.8	16.4	15.0
	CSM	11.0	11.5	11.5
Solar radiation (MJ/m^2^)	Rauscedo	18,593	20,130	19,853
	CSM	16,593	17,516	16,817

## Data Availability

Data is contained within the article or Supplementary Material.

## Supplementary Materials

The following supporting information can be downloaded at: https://www.mdpi.com/article/10.3390/plants13060816/s1, Table S1: Two-way ANOVA results on descriptive sensory analysis of the wines produced in the two areas (CSM and Rauscedo) in the two vintages (2011 and 2012); Dataset S1: Transcriptomic dataset; Figure S1: DEGs GO enrichment.
