# Supplementary material for: The Role of Terroir on the Ripening Traits of V. vinifera cv ‘Glera’ in the Prosecco Area
Source: Plants (Basel). 2024 Mar 12;13(6):816. doi: 10.3390/plants13060816 (PMC10975336; doi:10.3390/plants13060816)
Supplement: Supplementary file 1 [file plants-13-00816-s001.zip › Supplementary data.pdf]

Supplementary Table S1. Two-way ANOVA results on descriptive sensory analysis of the wines produced in the two areas (CSM and Rauscedo) in the two vintages (2011 and 2012). Attributes were quantified by a tasting panel formed by seven people using a ten-point intensity scale.

**Attribute**	***P*-value**		
	**Year**	**Site**	**Year × Site**
Olfactory intensity	0.101	0.780	0.962
Elegance	0.266	0.318	0.239
Rose	0.191	0.901	0.720
Lemon	0.294	**0.017**	0.060
Apple	0.851	0.575	0.851
Pear	0.587	0.860	0.550
Banana	0.838	0.934	0.832
Pineapple	0.736	0.940	0.411
Wisteria/acacia flower	0.840	0.640	0.672
Vegetable	0.343	0.189	0.274
Fresh vegetable	0.201	0.293	0.802
Fruity	0.192	0.308	0.354
Floral	0.362	0.520	0.943
Pleasentness	0.797	**0.044**	0.152
Acidity	0.101	0.380	0.457
Savouriness	0.405	0.455	0.378
Balance	0.071	**0.014**	0.071

Supplementary Figure S1. DEGs GO enrichment. Gene Ontology (GO) enrichment analysis performed on significantly differentially expressed genes (DEGs) in Rauscedo at mid ripening (A) and ripening (B) and in CSM at ripening (C). No statistically significant enrichment was found for DEGs featuring the CSM site at the ripening stage.

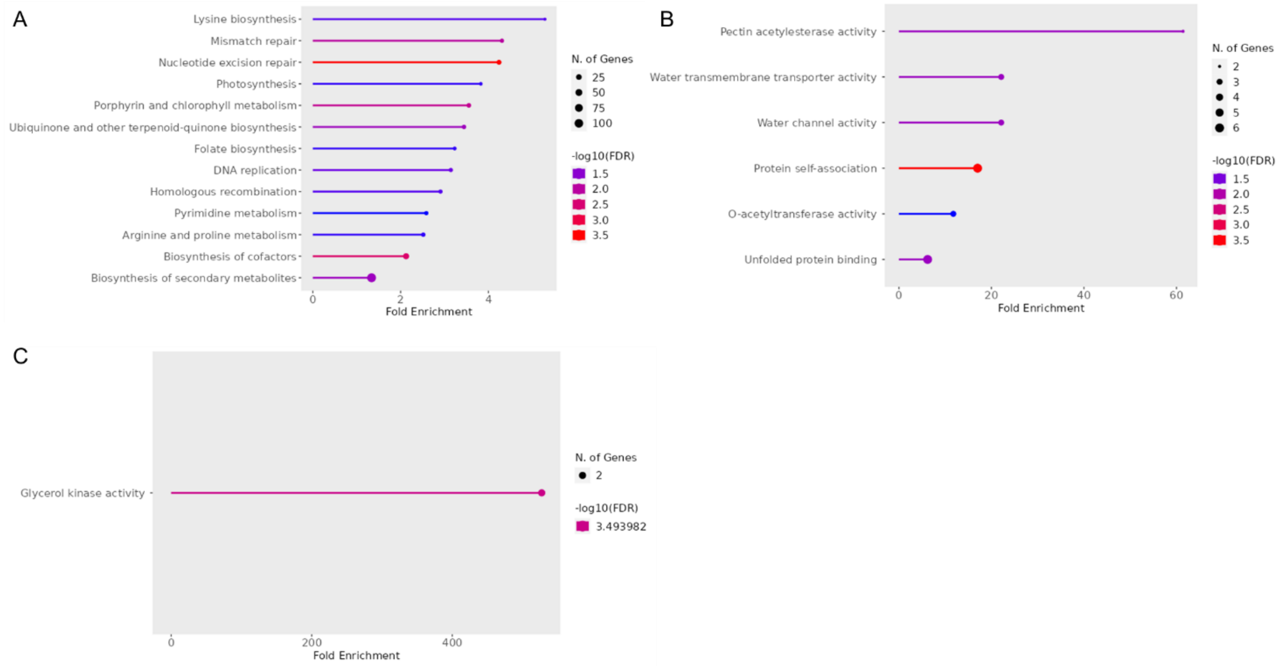

Supplementary Dataset S1. Transcriptomic dataset. Transcriptome of Glera grape berries collected in Rauscedo and Col San Martino (CSM) vineyards at mid ripening (MR) and ripening (R) stages (2012 growing season). For each transcript, the microarray Probe ID, the 12XV1 code, the VCOST.V3 code, the gene description, INTEGRAPE catalogue gene name, and the Fluorescence values (in triplicate for each sample) are indicated. Differentially Expressed Genes (DEGs; *t*-test; $p<0.01$) among vineyards at MR and R stage are indicated with an asterisk. The Fold change (FC) comparing the expression value of DEGs in the two experimental sites is also indicated.
